# Server-Focused Security Assessment of Mobile Health Apps for Popular Mobile Platforms

**DOI:** 10.2196/jmir.9818

**Published:** 2019-01-23

**Authors:** Jannis Müthing, Raphael Brüngel, Christoph M Friedrich

**Affiliations:** 1 University of Applied Sciences and Arts Dortmund Department of Computer Science Dortmund Germany; 2 Institute for Medical Informatics, Biometry and Epidemiology University Hospital Essen Essen Germany

**Keywords:** mobile health, mobile apps, data security, computer security, confidentiality, health information technology, servers, data protection

## Abstract

**Background:**

The importance of mobile health (mHealth) apps is growing. Independent of the technologies used, mHealth apps bring more functionality into the hands of users. In the health context, mHealth apps play an important role in providing information and services to patients, offering health care professionals ways to monitor vital parameters or consult patients remotely. The importance of confidentiality in health care and the opaqueness of transport security in apps make the latter an important research subject.

**Objective:**

This study aimed to (1) identify relevant security concerns on the server side of mHealth apps, (2) test a subset of mHealth apps regarding their vulnerability to those concerns, and (3) compare the servers used by mHealth apps with servers used in all domains.

**Methods:**

Server security characteristics relevant to the security of mHealth apps were assessed, presented, and discussed. To evaluate servers, appropriate tools were selected. Apps from the Android and iOS app stores were selected and tested, and the results for functional and other backend servers were evaluated.

**Results:**

The 60 apps tested communicate with 823 servers. Of these, 291 were categorized as functional backend servers, and 44 (44/291, 15.1%) of these received a rating below the A range (A+, A, and A−) by Qualys SSL Labs. A chi-square test was conducted against the number of servers receiving such ratings from SSL Pulse by Qualys SSL Labs. It was found that the tested servers from mHealth apps received significantly fewer ratings below the A range (*P*<.001). The internationally available apps from the test set performed significantly better than those only available in the German stores (alpha=.05; *P*=.03). Of the 60 apps, 28 (28/60, 47%) were found using at least one functional backend server that received a rating below the A range from Qualys SSL Labs, endangering confidentiality, authenticity, and integrity of the data displayed. The number of apps that used at least one entirely unsecured connection was 20 (20/60, 33%) when communicating with functional backend servers. It was also found that a majority of apps used advertising, tracking, or external content provider servers. When looking at all nonfunctional backend servers, 48 (48/60, 80%) apps used at least one server that received a rating below the A range.

**Conclusions:**

The results show that although servers in the mHealth domain perform significantly better regarding their security, there are still problems with the configuration of some. The most severe problems observed can expose patient communication with health care professionals, be exploited to display false or harmful information, or used to send data to an app facilitating further damage on the device. Following the recommendations for mHealth app developers, the most regularly observed security issues can be avoided or mitigated.

## Introduction

### Mobile Health Apps

The ubiquitous availability of the internet and mobile devices facilitates new and powerful applications. Mobile health (mHealth) is describing health care–related usage of mobile devices [1]. Although some apps offer user-tailored health information, others facilitate easier communication between patients and health care professionals or offer shop systems for medication sale. mHealth apps have an inherently higher need for security. Besides the importance to protect health data of patients that are collected using apps, by ensuring authenticity of the communication partner and confidentiality of data in transit, information coming from apps must also be protected from unauthorized changes to the content displayed (thus data integrity must be maintained). In contrast to browser-based websites or Web apps, the security of connections used by (native) mobile apps is not transparent to the user. Web browsers display both the use of a secure connection and, importantly, display issues with connection security prominently [2].

In earlier research, client-focused transport security concerns regarding mobile apps were studied [3]; this study focuses on the security of server infrastructure of mHealth apps.

### Server Security

The functionality of most apps relies on communication with a remote server over the internet. HTTP is the standard when it comes to client-server communication in the context of mobile apps [4] and offers no security features [5]. The communication through public infrastructure can potentially be observed, modified, or redirected. This endangers the integrity of data displayed by an app and confidentiality of the data sent to and received from a server and also could enable a malicious party to impersonate a server. Furthermore, a publicly reachable server must also guarantee availability [6].

The infrastructure between client and server represents an untrusted medium. Any third party in a privileged position between both communication partners can read and modify all data exchanged. A privileged position in this context is any hop on the path between them (bridges, routers, and gateways) [7]. A common attack vector is a technique called address resolution protocol (ARP) spoofing [7,8]. This enables the attacker to receive all requests normally intended for the router on the local network. Attacks where the third party is located between client and server and can read and modify messages are called man-in-the-middle (MitM) attacks. Another recent example enabling MitM attacks against clients of Wi-Fi Protected Access (WPA) 2 wireless networks is the Key Reinstallation Attack (KRACK) [9]. Older or unpatched protocols such as WPA and wired equivalent privacy (WEP) are also vulnerable to attacks, enabling traffic decryption or MitM setups [10,11]. For attacks on Wi-Fi infrastructure, physical proximity to the target is required. An attacker at least needs to be in the range of the victim’s access point. For the KRACK attack, the attacker needs to be close to the victim’s device.

To ensure confidentiality and integrity of data sent through an untrusted medium, the Transport Layer Security (TLS) protocol is used [12]. It is an important part of today’s internet security infrastructure. TLS was designed for authenticity, integrity, and confidentiality protection of the underlying communication channel by offering secure authentication, data integrity protection, and confidentiality through asymmetric and symmetric cryptography. TLS is situated in the application layer of the transmission control protocol (TCP)/ internet protocol (IP) stack and can wrap and secure HTTP connections. TLS-secured HTTP connections are called HTTP Secure (HTTPS) connections [13].

Given certain server and client configurations, TLS can be set up to offer forward secrecy [14]. This means if secret keys are compromised in the future, past communication stays secure and cannot be decrypted with the compromised credentials alone.

Without this secure wrapper authenticity of a server, confidentiality of information exchanged with a server and the integrity of data sent to an app cannot be guaranteed, and the app might display arbitrary text, pictures, or video data. Because an attacker can potentially feed an app arbitrary input, missing integrity can also be exploited to make the app decode an image, video, or other data that could exploit vulnerabilities in decoders. The Stagefright exploit on Android devices, for example, relied on Android operating system (OS) processing modified media files [15].

TLS and its predecessor the Secure Socket Layer (SSL) protocol are not without security flaws. Since its introduction, multiple vulnerabilities in different layers of the protocols or implementations of the protocols were found and exploited to undermine their security [16]. To keep a server (and therefore patients) secure, special scrutiny and vigilance regarding new threats are required from server operators [17]. It is crucial to react quickly to the publication of new vulnerabilities. A malicious third party only has to test the exploitability of all known vulnerabilities to find a way to attack the server-client communication. Some well-known examples are the Padding Oracle On Downgraded Legacy Encryption (POODLE), Heartbleed, and the recent Return Of Bleichenbacher's Oracle Threat (ROBOT) that was made public in December 2017 [18-20]. To address newly found security issues in SSL and its successor TLS, new versions of the protocol are released regularly. Use of a newer version protects from known security flaws of older versions.

TLS relies on digital certificates to authenticate a server to clients [12,21]. These certificates must be issued (and signed) by certificate authorities (CAs) and have multiple characteristics that must be checked for a certificate to be valid for a given domain. Some characteristics are that a certificate must (1) be issued for the domain requested, (2) have a *valid from* date and must be before the current date, (3) have a *valid until* date in the future, and (4) must not be revoked.

A certificate’s revocation status can be checked against a CA’s certificate revocation list. As CAs are the roots of the chains of trust, they must operate responsibly. There are some CAs that offer certificate services for free (eg, the Linux foundation’s *Let’s Encrypt*), whereas others charge for certificates issued by them [22]. The management of the server certificates and safekeeping of corresponding private keys are also crucial to the security a server can offer.

A general overview of security threats to internet-connected systems can be found at the Open Web Application Security Project (OWASP) [23]. The project collects information, tools, and best practices to avoid common security issues. The OWASP Top 10 and the OWASP mobile Top 10 are of great relevance to the research presented here [24,25]. Although not all vulnerabilities are relevant, the lists were a valuable starting point for the design of the tests.

### Prior Work

In prior research, the transport security of mHealth apps from a client’s perspective was investigated [3]. The research inspected the data exchanged between iOS and Android client apps and a server and evaluated it under security considerations. This also included the use of TLS and the TLS version. Furthermore, it considered the validation of server certificates by the clients. The study found severe problems with 40% of all 53 tested apps.

Existing literature also evaluated metadata of mHealth apps from Google’s Play Store and Apple’s App Store [26]. The study did not perform tests or technical analysis. A study of popular mobile apps from China found 97% of apps surveyed provided no information security [27]. The authors limited their investigation to the evaluation of available documentation and availability of auditing reports.

Other research focused on 22 mHealth apps and found that 18 of those apps send data unencrypted over the internet [28].

As mobile apps are not limited to stock HTTP(S) implementations, the study of HTTPS implementations in Android apps is relevant [29]. The publication discusses common flaws in TLS deployments and server configurations.

In the realm of the Internet of Things, existing research analyzed internet-connected toys for children in regard to security and privacy concerns [30]. The authors also bring attention to severe transport security issues of toys when communicating with their backend servers.

Servers have been in use for many years to serve websites and interfaces for internet-based services. The servers’ purpose to supply mHealth apps with data and functionality is only one of their most recent use cases. Due to their inherent exposure to public infrastructure (the internet), they always offered an attractive attack surface. As a consequence, knowledge about problems and secure configuration of servers is widely available [5,6,31,32]. An overview of the general landscape of SSL/TLS security on the internet can be observed on Qualys SSL Lab’s SSL Pulse website [33].

mHealth apps are getting attention by the media and are covered in regard to treatment of patient data [34]. There is an initiative to build a central place to rate apps in regard to privacy matters (among other criteria) [35]. PrivacyScore has similar objectives and offers a configurable interface to test for a number of security and privacy issues of websites [36].

Existing research mostly considers nontechnical characteristics of mHealth apps, client-side implementations of apps, or solely the use of any encryption at all. In contrast, this research will focus on the configuration of servers used by mHealth apps.

The Methods section will describe how the tested apps were selected. Furthermore, it presents and explains transport security issues for servers and lists the tools used to test for these issues. In the Results section, the tested apps are presented. The test methodology is explained before the aggregated results are listed. These results will be discussed, and common issues will be pointed out.

## Methods

### App Selection

In prior research, free apps from 3 different European app stores were selected. As differences in behavior between apps from different European countries were not found without loss of generality for this study, only free apps from the German app stores’ top lists were chosen [3]. Many apps from the German top-downloaded lists are popular across other nations’ app stores. The difference between internationally available and popular apps and apps only in the top lists of the German stores will be discussed in the Results section. To mitigate any platform-dependent bias, apps for Android and iOS are tested.

### Relevant Server Security Considerations

HTTP by itself transmits information as clear text without any encryption. It is an application layer protocol and can be secured by being used on top of a secure TLS connection [4,12,13]. TLS and its predecessor SSL are designed to ensure the authenticity of communication partners, confidentiality between parties, and integrity of transmitted data. To achieve this, TLS uses asymmetric cryptography and public key infrastructure (PKI) for authentication and exchange of key material. Symmetric encryption is used for payload data encryption [37,38].

SSL and TLS use version numbers. As earlier versions of the protocol had serious security issues, this paper will take the version of SSL or TLS into consideration [16,18]. While SSL 2.0 is considered insecure because of structural vulnerabilities [39], the POODLE exploit enables third parties to recover plaintext from SSL 3.0-protected traffic [40,41]. Apart from SSL 2.0 and SSL 3.0, newer TLS versions do not have known security vulnerabilities if the server (and client) is properly configured. Lacking a proper configuration, older versions such as TLS 1.0 are vulnerable to an improved POODLE attack and other vulnerabilities [18]. Another TLS 1.0 vulnerability can only be efficiently mitigated by the clients: the Browser Exploit Against SSL/TLS (BEAST) [42]. Although most modern browsers do mitigate the issue, the security of the protocol is still not controllable on the server side. TLS 1.1 and later protocols are not vulnerable to such attacks and can be configured on the server side to use secure ciphers [43]. More recent versions include improvements that are considered more secure. The use of SSL/TLS and the lowest supported version number will be part of the evaluation. We also evaluated support for the recently approved TLS 1.3 (August 2018) and mentioned it in the Results section [44].

To ensure the use of HTTPS and prevent protocol downgrade attacks, HTTP Strict Transport Security (HSTS) can be used [45]. A downgrade attack is designed to get a client to connect to the server using an unsecured HTTP connection. This enables a malicious third party to perform a MitM attack and consequently read and modify sensitive information. For HSTS to be used, the server sends a special header in response to a request. This tells the client to only connect through secure HTTPS connections. For HSTS to work, both client and server have to support it. Although HSTS is most important for browser-based Web apps, many apps include Web components that use platform browser components. In addition, Web-based versions of apps often exist. The presence of HSTS headers in server responses will be listed in the Results section.

To provide a better understanding of the following considerations, a basic understanding of the TLS handshake is required [12]. This description will focus on the most common case of server authentication only.

The client initiates the connection by sending a *client hello* message. This includes its highest supported TLS version, a random value, suggested compression methods, and its supported cipher suites. The server, in turn, answers with the chosen protocol version, cipher suite, compression method, and a random value. In a *certificate message*, the server also sends its certificate. The client now creates a premaster secret, encrypts it with the server’s public key, and sends it to the server. Both parties generate the master secret and session keys based on the premaster secret. The client sends a *change cipher spec* message to the server to inform the server that it will use the session key for hashing and message encryption. This is followed by a *client finished* message. The server receives this message, switches to symmetric encryption for further messages, and sends a *server finished* message.

During the TLS handshake, the client uses the servers’ public key from its certificate to encrypt a premaster secret. The encryption algorithm is dependent on the negotiated cipher suite. The certificate sent by the server fits the negotiated cipher suite: if, for example, a cipher suite is chosen that includes the *Elliptic Curve Diffie Hellman Ephemeral* (ECDHE) algorithm for key exchange, the certificate must include an elliptic curve (EC) public key [46,47]. As explained in the TLS handshake, the security of the key exchange is essential for the security of the connection. If an adversary is able to decrypt the *premaster* key by brute force, the security of the TLS connection would be compromised. To make this harder, the algorithm used as well as the key length of the public key are important. A commonly used algorithm is the Rivest-Shamir-Adleman (RSA) algorithm [48]. Key sizes vary from 1024 to 4096 bit. It has been shown that a 1024 bit key does not offer sufficient security [49]. Moreover, 2048 bit is the commonly recommended lower limit for RSA keys. The added complexity and negative performance impact during the TLS handshake are disadvantages of the use of longer keys. Newer algorithms such as the EC algorithm do require smaller keys, less computational requirements for clients, and servers while offering equivalent security [47,49]. The key algorithm and length are part of this evaluation.

Another aspect related to the handshake is the selection of the cipher suite. A server has a number of supported cipher suites [12,50]. When the client sends its list of possible cipher suites, the server selects one it supports. The most secure cipher suite should be negotiated between client and server. A server can be configured to have an order of preference for cipher suites [17]. If present, the server will choose the suite highest in priority, which is supported by the client. Whether a server has a preferred order is part of the results of the study because of the importance of the chosen cipher suite for the encryption of user traffic.

For the same reason, the list of supported cipher suites will be considered. Although uncommon, in the worst case, cipher suites may define no encryption at all for the TLS traffic. Other cipher suites define algorithms that can be cryptographically attacked and should not be used anymore. This section does not list all ciphers and their vulnerabilities, but the use of insecure cipher suites is part of the evaluation.

The authentication of the server is based on the server’s certificate [12,21,51]. A certificate must fit certain criteria to be considered valid by the client. It must be issued for the requested domain, must not be expired or revoked, and must be trusted. In a PKI, a client trusts root certificates issued by CAs. These CAs use the corresponding private key to sign certificates of servers (or other sub-CAs). When the client verifies the validity of a server certificate, it follows this chain of trust from the server certificate until one of the certificates in the client’s trusted root certificates is referenced. Certificate (chain) issues are also part of the research performed for this study.

Older SSL and TLS versions are vulnerable to certain exploits undermining their security. In addition to vulnerabilities of older versions, implementation-dependent issues such as Heartbleed and others are relevant. Heartbleed is an issue in the popular OpenSSL cryptographic software library. It enables an attacker to read memory contents from the server if the library is not patched. Another recently (December 2017) discovered attack uses an issue in RSA implementations and makes the key exchange observable by attackers (ROBOT) [19]. A server that supports RSA for its key exchange, and that is using a vulnerable implementation, is at risk to be attacked [52]. A relative of the BEAST vulnerability discussed earlier also enables attacks against TLS 1.2. It is named the Compression Ratio Info-leak Made Easy (CRIME) and works in conjunction with the use of cookies by protocols that use data compression (such as HTTPS) [53]. It can be used to observe and use a client’s authentication cookie to enable further attacks. The vulnerability can be counteracted on the client as well as on the server side. The Browser Reconnaissance and Exfiltration via Adaptive Compression of Hypertext (BREACH) attack is a variant of CRIME and can be exploited to a similar effect [53]. A comprehensive overview of known attacks against TLS can be found as a Request For Comments (RFC) by the Internet Engineering Task Force (IETF) [53]. Vulnerability to known attacks is considered during the tests.

Because the physical location of a server has consequences for applicable law, the server location is considered in this study. Specifically, this study lists whether servers are inside the European Union (EU), as it is considerably harder for service providers dealing with data belonging to European citizens to host these data outside the EU and still comply with the General Data Protection Regulation (GDPR) [54,55]. Explicit exceptions are GDPR-compliant servers outside the EU covered under the EU-US privacy shield [54,56]. As mentioned in the Limitations section, this study does not differentiate GDPR-compliant servers outside the EU, and servers are simply listed to be located outside EU territory.

### Selection of Appropriate Test Tools

The first step of the tests is to find out which of the apps communicate with which backend. Similar to prior research, the BProxy tool was used to facilitate the first test phase [3,57]. This software acts as a proxy between a device running an app and the internet. It is open source and can be found on GitHub [57]. All traffic from the device is analyzed by the proxy, and a report is displayed. As this study is concerned with server-side transport security aspects, only the domain names of servers an app communicates with and the use of unsecured connections are of interest. As the proxy records all traffic to and from the device, filtering is necessary to find out which domains are part of the app’s backend. Traffic was inspected to distinguish between app-related servers and other servers. To facilitate correct categorizations, the disconnect service was used and the results were inspected manually, similar to the methodology of a prior publication [3,58].

To test the servers behind the domains, the testssl script and the Qualys SSL Labs test suite were chosen [59,60]. The testssl script uses OpenSSL to perform tests against a targeted server from the local machine. It generates a comma-separated value (CSV) file containing all findings for the domain. The script checks for some relevant characteristics, including all described relevant concerns. The script is actively developed and maintained to contain tests for recently discovered vulnerabilities. At the time of testing, the ROBOT attack was relatively new and already included in the development version of the testssl script [19,59]. The SSL Labs test suite is a Web-based tool for SSL/TLS-related tests. Qualys SSL Labs also offers a command line reference implementation for test automation [61]. The results include similar attributes as the testssl script but importantly assign a graded score A to F. This score is the result of an automated evaluation of the characteristics and vulnerabilities observed. A guide on how this score is calculated and, consequently, how SSL Labs rates the severity of security characteristics can be found on GitHub [62]. This rating guide is updated by Qualys on a regular basis, and a changelog can be found as part of the guide mentioned previously. The rating consists of multiple considerations and includes all but the first item in Table 1: (1) validity and trust of the certificate used by the server, (2) supported protocols (SSL 1.0 up to TLS 1.3), (3) key exchange algorithms supported (older algorithms score lower because of security issues), and (4) cipher suites supported (if no secure, up-to-date cipher suites are supported, the grade will be lower).

Each category scores between 0 and 100. The scores are combined, which results in a single overall score for the server. A 0 in any category will result in an overall score of 0. Although a low score results in an overall lower result, a 0 in a category is indicating a fatal security issue. An example for the score calculation in the supported protocols category according to the Qualys SSL Labs’ server rating guide looks like this:

1. Start with the score of the best protocol.
2. Add the score of the worst protocol.
3. Divide the total by 2 [62].

Table 2 lists the scores that Qualys SSL Labs assigns each supported protocol version at the time of writing. A 0 in this category, for example, can only occur if only SSL 2.0 was supported. As this is a long outdated and insecure version, a score of 0 is justified. The other categories are evaluated in a similar manner.

**Figure 1:**
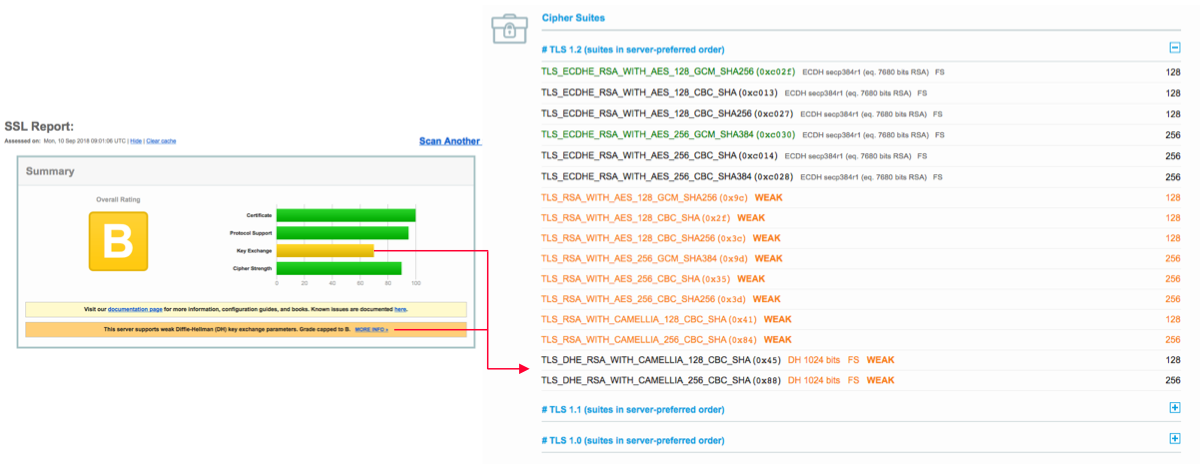
Exemplary rating and scores of a domain in the test pool. The server was downgraded mainly for offering weak Diffie-Hellmann key exchange. The scores in the distinct categories can be observed to the left. On the right, the offered cipher suites, which include the key exchange algorithms, are listed and marked as weak points.

Server configurations that cannot be captured by a score are accounted for by special rules to correct the grade calculated [62]. An example of a server rated B and the scores in each category can be observed in Figure 1.

**Table 1:** Main considerations evaluated in this study.

Security considerations^a^	Description
Use of secured connections (SSL^b^/TLS^c^)	The use of any unsecured connections
SSL/TLS version	Evaluating the supported versions of SSL/TLS
Key exchange support	The cryptographic algorithm used to exchange the keys during the handshake for the following symmetric encryption
Cipher support	The cipher negotiated between client and server dictates what symmetric encryption is applied after the handshake and key exchange
Certificates	The security characteristics TLS offers rely on the server’s certificate. Any trust issues here are critical
Vulnerabilities	Certain attacks are based on specific implementations or the absence of a patch on the server
HSTS^d^	Support HSTS can prevent downgrades to HTTP

^a^All but the first one (use of unsecure connections) are tested for by the tools presented in later sections.

^b^SSL: Secure Socket Layer.

^c^TSL: Transport Layer Security.

^d^HSTS: Hypertext Transfer Protocol Strict Transport Security.

**Table 2:** Qualys SSL Labs scoring for protocol support.

Protocol	Score (%)
SSL^a^ 2.0	0
SSL 3.0	80
TLS^b^ 1.0	90
TLS 1.1	95
TLS 1.2	100

^a^SSL: Secure Socket Layer.

^b^TLS: Transport Layer Security.

The overall score, based on a constantly updated set of rules, and the added safety of 2 sources for server security assessment are the reasons why both testssl (Version: 2.9.5dev) and the Qualys SSL Labs suite (Version: 1.32.6) are used. To determine the physical location of a server, a Web-based service was used [63,64]. To aggregate the results, a consistent logic was chosen. For negative observations (such as a rating below the A range containing ratings A+, A, and A− from Qualys SSL Labs), the worst observation in a given category was recorded. For positive observations, the inverse logic was applied: a category of an app was counted as supporting the positive characteristic (such as HSTS and TLS 1.3) when at least one server supported the feature.

### Limitations

The tests performed as part of this research focus on transport security and weaknesses in TLS configurations on the server side. The research did not include a full penetration test for every server that was found. Although availability is an important characteristic for a server, this attribute was not part of our tests as testing for denial-of-service (DOS) hardening would require staging test DOS attacks [6]. This would not be legally possible without cooperation of every app developer [65].

As this study focuses on the server side, correct certificate validation in the apps is not tested, neither is the traffic analyzed for leaked information of any kind.

Apps that did not function normally made a test of functions impossible and were removed during app selection. This includes apps that prevented traffic inspection by our test tool. It is possible that these apps used certificate pinning to ensure the servers identity.

The app tests were performed manually. Complete functional coverage cannot be guaranteed. Especially, functionality behind paywalls (in-app purchases or similar facilities) remained untested.

Categorization of servers as functional backends or others was aided by a Web-based service that keeps a list of known advertising and tracking servers but, in part, had to be performed manually [58]. The categorization in uncertain cases was vigilantly checked but might still contain errors.

The location of a server is hard to pin down. It is possible for a server to be inside the EU, although our test using a Web-based service did state a different location. Servers are often used to answer requests from different locales. A request from within the EU might be answered from a server in Ireland, whereas a request from the United States might be handled by a server located in the United States.

A server based outside the EU might fall under the EU-US privacy shield [56]. This allows organizations to store data outside the EU and still comply with applicable EU law [54]. As there is no straightforward way known to the authors to check if a server is part of privacy shield or complies with the GDPR in other ways, this differentiation is not made in this study.

A correct certificate validation depends on a dependable PKI. Research has shown that domain name system (DNS)–based domain validation by CAs is not always dependable and can be abused to have a CA issue certificates for arbitrary domains [66,67]. An attacker in possession of a valid certificate for the domain name requested can impersonate an authentic server even when the client applies correct certificate validation. As this is a CA’s responsibility and an app provider has no control in this regard, this issue will not be addressed further in this study.

## Results

### App Selection

To select a sample set of mHealth apps, the top-downloaded lists of free apps from the Android and iOS app stores are considered as a starting point. The *medical* category was selected as it is most likely to contain mHealth apps. In previous research, similar client security problems in apps from 3 different countries were found [3]. Differences in behavior between apps from stores of different countries were not found. In this study, 30 apps from the German Google Play store and 30 apps from the iOS AppStore are considered (60 apps in total). The top-downloaded lists were generated from the app store analytics site AppAnnie on August 31, 2018 [68]. Top lists for a specific day are available after registration on the website. The *medical* category includes apps that fulfill a broad spectrum of functions. For this reason, apps from both app stores were categorized. All subcategories found in the *medical* categories are listed and defined in Multimedia Appendix 1. The 5 subcategories selected in this study are (1) fertility, pregnancy, and parenthood; (2) drug information, shopping, and compliance; (3) reference and learning; (4) consultation, communication, and interaction; and (5) health, fitness, and monitoring.

To improve variety, the 6 highest positioned apps from these categories and both lists were selected. If a selected app was untestable with test devices, the next most popular app of the same category was selected. This was the case for 9 apps. Of all 60 apps, 26 (26/60, 43%) were in at least one other top 500 list in France, the United Kingdom, or the United States, covering a portion of internationally relevant mHealth apps. The top positions in the stores, developer information, and subcategorization of the apps are visible in Multimedia Appendices 2 and 3.

### Performing the Tests

To help parallelize the app testing, iOS and Android apps were tested separately. All apps for both platforms were downloaded from their respective app stores and installed on the devices (iOS 11.4.1 on an iPhone 7 and Android 6.0.1 on a Nexus 7). Before any test, the apps were stopped and restarted. As described previously, the devices are set up to use an HTTP proxy for all HTTP/S traffic. The BProxy tool was used to compile a list of relevant domains.

Many apps communicate with a plethora of endpoints. In addition, the Android or iOS OSs also communicate through HTTP/S connections for multiple purposes, including mail fetching, checking for updates, and sending analytics data. To filter the domains observed during the test of an app, multiple app runs are used to try to distinguish between app traffic and *background* traffic not related to the app. In addition, manual filtering was performed. Service calls from the OS (such as mail server communication) were disregarded. When necessary, an account for the app was created and activated for the tests.

A bash script was used to sequentially test each remaining domain using the testssl script [59]. The Qualys SSL Labs Server Test Web-based tool offers a bulk application programming interface (API) to test multiple domains. Both tools return JavaScript object notation formatted files [59,69]. To reach the objective to generate informative and easy-to-understand results, it was decided to distinguish between backend, advertisement or tracker or analytics, external content, and other servers. The functional backend category includes servers that directly work to supply the app with functional content, perform operations with input from the app, and seem to be under the control of the app developer. All other servers were sorted into the second category of *other* servers, including all servers that serve advertisements, track user activity, and/or are part of an analytics service supplying the app provider with information about app usage. The categorization was performed by evaluating the domain name and analyzing HTTP/S traffic using the BProxy. Categorization as an *other* server was improved using the disconnect service where 311 of the 532 (58.5%) *other* servers were listed [58].

**Figure 2:**
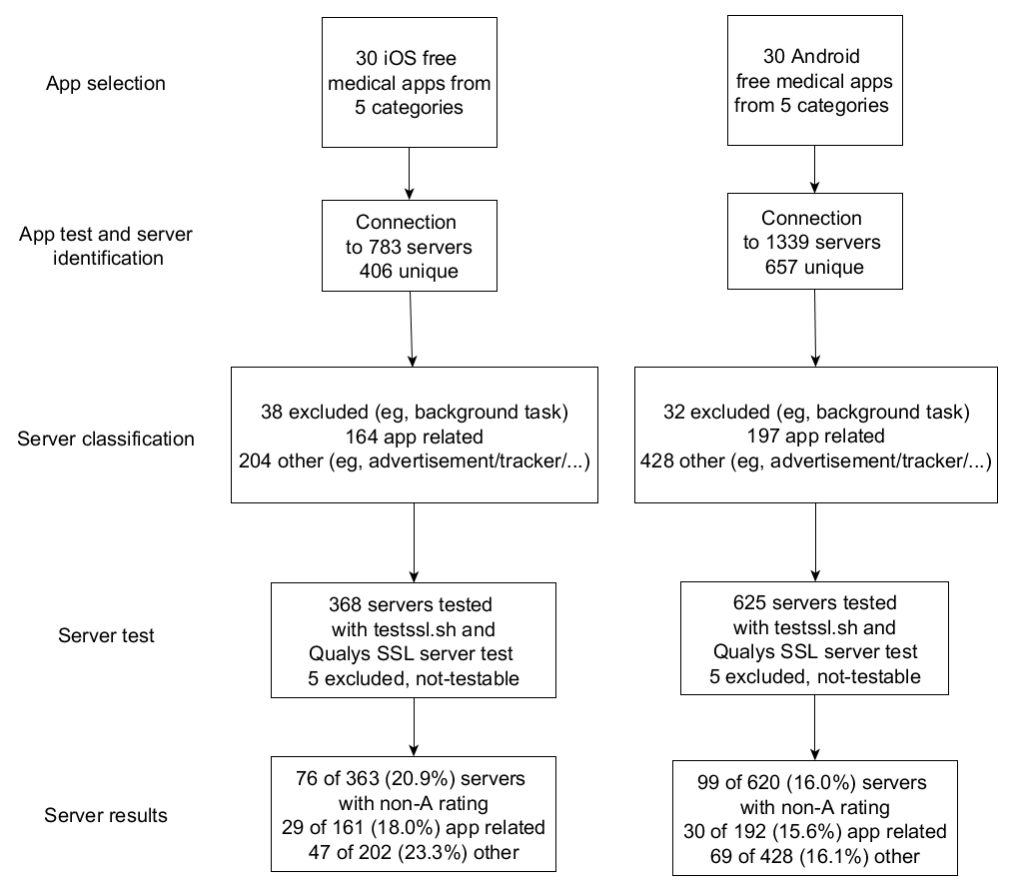
Workflow for tests of mobile health apps. In the app selection phase, the 6 most popular apps from each of the 5 subcategories were selected. In the app test and server identification phase, the traffic between apps and servers was observed and unique servers recorded. The servers were categorized or disregarded as facilitating irrelevant background tasks (server classification phase). The relevant servers were used as the input for the testssl script and the Qualys SSL Labs suite (server test phase). Finally, the results tables were compiled (server results).

Figure 2 shows a graphical representation of the workflow described. To help evaluate and compile result summaries, the R programming language (version 3.4.2) was used [70]. Negative and positive observations were collected separately. During the compilation of the summaries, a negative or positive observation for a server during the tests was counted as the results for the entire *functional* or *other* backend category of the app. For some analyses, these sets have been combined to obtain results for medical apps in general.

### Summarized Results

The 60 apps tested communicated with 823 different servers. The distribution of the number of servers apps communicated with can be observed in Table 3. All apps communicated with servers beyond their functional backend. The median number of other servers the apps across both platforms communicated with is 18.5. The median number across Android apps is more than twice (24.5) the amount in comparison with iOS apps (11). In the most remarkable case, an Android app communicated with 82 *other* servers beyond its functional backend.

For easier evaluation of the overall results, the findings were divided into positive and negative results and summarized in Tables 4 and 5. These list the results for both functional as well as other backends. As the functional backends serve requests directly related to an app’s functionality, these are the most interesting results.

Detailed results of the security tests can be found in Multimedia Appendices 2 and 3. They list the findings on a per app and per function basis. For further details, please refer to Multimedia Appendices 4 and 5. The tables in these appendices contain the number of connections per apps to servers exhibiting the security characteristics that are part of this study.

**Table 3:** Minimum, maximum, and median numbers of functional and other backends for iOS and Android apps.

Statistics	Android (functional)	iOS (functional)	Android (others)	iOS (others)	Overall (functional)	Overall (others)
Minimum number of servers	0	1	2	1	0	1
Maximum number of servers	33	21	82	39	33	82
Median number of servers	5	4	24.5	11	4.5	18.5

**Table 4:** A summarized table of negative results regarding backends of Android and iOS apps. Negative observations are counted for the functional or other category on a per-app basis when it was present in at least one of the apps’ servers.

Security issues	Android (functional), n=30	iOS (functional), n=30	Android (others), n=30	iOS (others), n=30	Total (functional), n=60, n (%)	Total (others), n=60, n (%)
Qualys SSL Labs non-A rating	14	14	24	24	28 (47)	48 (80)
Server only offers TLS^a^ version <1.2	5	3	0	1	8 (13)	1 (2)
Server without set cipher order	7	5	4	1	12 (20)	5 (8)
Certificate (chain) validation issues present	9	5	14	6	14 (23)	20 (33)
Downgrading vulnerabilities	5	4	8	7	9 (15)	15 (25)
Servers outside the EU^b^	24	21	30	30	45 (75)	60 (100)
Missing forward secrecy support	2	2	1	1	4 (7)	2 (3)
Unsecure connection/s observed	10	10	10	8	20 (33)	18 (30)

^a^TLS: Transport Layer Security.

^b^EU: European Union.

**Table 5:** A summarized table of positive results regarding backends of Android and iOS apps. One observation of a positive characteristic makes the functional or other category count for the app.

Positive findings	Android (functional), n=30	iOS (functional), n=30	Android (others), n=30	iOS (others), n=30	Total (functional), n=60, n (%)	Total (others), n=60, n (%)
TLS^a^ 1.3 support observed	4	5	21	17	9 (15)	38 (63)
HSTS^b^ support observed	12	15	28	25	27 (45)	53 (88)

^a^TLS: Transport Layer Security.

^b^HSTS: Hypertext Transfer Protocol Strict Transport Security.

Of the apps tested, 28 (28/60, 47%) used servers where at least one functional backend received a non-A rating from Qualys SSL Labs. In contrast, 48 (48/60, 80%) apps used advertisement, analytics, or external content providers (others) that received a rating below the A range.

Regarding the support of TLS 1.2, only 8 (8/60, 13%) apps used functional backend servers that did not offer TLS 1.2 (3 iOS apps and 5 Android apps). All but one app used only other servers that offered TLS 1.2.

Functional backend servers without a set cipher order were observed when using 12 (12/60, 20%) apps. Other servers without a cipher order were used by 5 (5/60, 8%) apps.

It was found that 14 apps (14/60, 23%) used functional backends that did not offer a valid certificate for the domain requested. This also includes certificates that fail proper validation for any reason (certificate chain issues and domain name mismatch). This issue was also found with other servers for 20 (20/60, 33%) apps.

It was discovered that 9 (9/60, 15%) apps worked with functional backend servers that were downgraded by Qualys SSL Labs because of their vulnerability to an exploit. More apps communicated with other backends that were downgraded (15/60, 25%) apps. Vulnerabilities that lead to a downgrade are related to the support of certain cipher suite or unpatched implementations on the server side [62]. Vulnerability to the POODLE attack was the most observed issue that led to a downgrade. The tables in the Multimedia Appendices 2 and 3 can be consulted for further information.

During the tests, it was found that 45 (45/60, 75%) apps appeared to use some functional backend servers outside the EU. The same is true for all 60 (60/60, 100%) apps regarding other backends.

Forward secrecy was supported by all but 4 (4/60, 7%) apps in at least one functional backend server. The support was observed in at least one other backend server for all but 2 (2/60, 3%) apps.

During the tests, we also recorded entirely unsecured connections by the apps to their servers. This was observed in 20 (20/60, 33%) apps during their communication with a functional backend server and in 18 (18/60, 30%) apps with *other* backend server.

During the evaluation of the test data, some apps showed especially severe issues and are responsible for many of the concerns listed in Table 4. Moreover, 1 app provider, for example, offered 4 apps in total (2 iOS and 2 Android apps). All these apps communicated with backends that did not support an up-to-date TLS version. In addition, it was found that the apps used a mix of unprotected HTTP and protected HTTPS connections to communicate with their backends, further undermining the security of the communication.

It was found that of the 291 functional backend servers, 15.1% received ratings below the A range by Qualys SSL Labs. Of the 532 nonbackend servers tested, 18.8% were rated below the A range. Qualys SSL Labs’ SSL Pulse website lists popular security characteristics of servers. They summarize these and also show the percentage of servers receiving a rating below the A range. In the statistics from September 2018, of the 139,849 servers, 37.75% received non-A ratings. A conducted chi-square test (alpha=.05) shows the tested servers of mHealth apps to be significantly better rated than servers observed by SSL Pulse in general (*P*<.001 for both functional backends as well as others) [33].

Chi-square tests were also conducted between each of the subcategories regarding the number of apps that communicated with servers receiving a non-A rating. For these statistical tests, iOS and Android apps as well as functional and other servers were not differentiated. It was found that *reference and learning* apps received a significantly worse rating when tested against both *fertility, pregnancy, and parenthood* (alpha=.05; *P*=.02) and *drug information, shopping, and compliance* (alpha=.05; *P*=.01) apps. The other categories showed no significant differences between each other. Looking at differences between internationally available apps and apps only present in the German top lists, the international apps were found to perform significantly better (alpha=.05; *P*=.03). For this test, any app that was also listed in a top 500 list in the United States, the United Kingdom, or France was considered international.

Looking at regularly contacted domains that can be observed in Table 6, the services behind these top 10 most contacted servers receive data from many of the apps tested and will be able to reconstruct a comprehensive user profile.

Many advertising and analytics companies operate multiple second-level domains. Using the disconnect.me lists revealed, a high number of domains requested belonging to Google’s and Facebook’s services [58]. Google is very much present in the overview. Almost all (55/60, 92%) apps communicated with servers under a Google API domain, and 8 of the 10 most frequently observed second-level domains are attributable to the company.

Of the 60 apps tested, 17 offered a user-controllable opt-out option for certain advertisement or tracking services. In earlier research, these options were mentioned as desirable [3].

As for positive observations, TLS 1.3 support was observed in 9 (9/60, 15%) apps when communicating with their functional backend servers and 38 (38/60, 63%) with other backend servers. HSTS support was observed in at least one functional backend server for 27 (27/60, 45%) apps and at least once for other backend server in 45 (45/60, 75%) apps. The high percentages in the *others* category is partly a result of the way the results were counted. The number of other servers was regularly higher than the number of functional backend servers, and the positive observations were combined using the Boolean or-conjunction. To gain further insight into how many servers of an app’s backend were exhibiting which security characteristic, please refer to Multimedia Appendices 4 and 5.

**Table 6:** Number of apps that communicated with a subdomain of the second-level domains listed.

Domain	Apps, n=60, n (%)
*.googleapis.com	55 (92)
*.google-analytics.com	46 (77)
*.google.com	38 (63)
*.googleapis.com	37 (62)
*.doubleclick.net	36 (60)
*.gstatic.com	33 (55)
*.crashlytics.com	29 (48)
*.google.de	23 (38)
*.googleadservices.com	23 (38)
*.fbcdn.net	11 (18)

## Discussion

### Principal Findings

Some backends display problematic characteristics. The most problematic cases exhibit invalid server certificates. In these cases, a client cannot distinguish between a real server certificate and a certificate from a third party attempting a MitM attack. The use of outdated TLS protocol versions can lead to integrity, authenticity, and confidentiality issues with data displayed or sent to the server. The implications vary from app to app. Affected apps did facilitate medical and drug information and interaction checks and patient to health care professional communication. The latter case can expose patient information and enable fabrication of answers from the health care professional to the patient.

In contrast to browsers, apps on mobile platforms have a disadvantage: they can (and do) hide transport security issues from their users. Although private data might be sent to a server without properly securing the connection, a developer can choose to ignore the issues. A browser, on the other hand, displays security warnings when a website can only be connected to using unsecure connections and will make interactions harder for the user [71]. Apple’s App Transport Security effort is a step in the right direction but will still allow exceptions for app-specified URLs [72]. It could be the objective of further work to evaluate the possibility for a mobile OS to monitor all traffic from an app and warn the user about insecure connections.

Apps that are localized, available, and successful in more than the German app store suggest bigger organizations with more resources. These organizations can be expected to also devote more resources to the (security) maintenance of their servers. The observation that internationally popular apps performed significantly better validates this expectation.

As discussed in the study by Müthing et al [3], the use of advertisement and tracking services in medical apps can pose as a challenge. These services offer app developers insight into the usage of their app. But the data are also collected by these services for further monetization and data mining [73]. As medical (patient) data are protected under special jurisdiction and should be protected for ethical reasons, the use of any third-party services must be met with scrutiny. For many apps, the tests revealed the use of a great number of servers from various services. The high number of apps that use the same services for advertising or analytics can be problematic. These services can collect user information across multiple apps [73,74]. Another reason to be aware of the use of third-party services is the relatively large number of servers used for advertisements or tracking that received a non-A rating from Qualys SSL Labs (48% of all apps).

Looking at the results in regard to server locations, all (60/60, 100%) apps used *other* servers outside the EU. When looking at the source data, it can be observed that these servers are often related to tracking, analytics, and advertising.

The data also reveal severe issues not directly visible when only considering the server setup: apps were observed using entirely unsecured connections or a mixture of secured and unsecured connections in communication with the same server. Although the backend server might be set up to use state-of-the-art TLS and certificates, this will always undermine potential security and put user data at risk.

The wide support of forward secrecy (functional: 15% and others: 63%) can be seen positively, as it prevents unauthorized decryption of sensitive data in the future and entails a performance burden for attacks on the connection. The high number (functional: 45% and others: 88%) in servers supporting HSTS can help to mitigate against protocol downgrade and cookie hijacking attacks.

During an earlier phase of the preparation of this study—and before the tests discussed so far—40 mHealth apps were tested. The 20 most popular apps from the *medical* category from the German iOS and Android app stores were tested with a very similar methodology as was described in this study. A description of the 40 apps, their categories, and the summarized results can be found in Multimedia Appendix 6. Detailed results per app tested for Android and iOS can be found in Multimedia Appendices 7 and 8, respectively. Some apps were tested in both test runs. Although the earlier results showed a lower number of functional backends receiving a Qualys SSL Labs grade below the A range (28% in early 2018 vs 47% in September 2018), there are also positive developments observable. Vulnerability to the ROBOT attack was observable in a nonfunctional backend in early 2018 but was fixed in the later tests. The relative rise of backends receiving a rating below the A range might be caused by a difference in classification but can also be attributed to the changes in the Qualys SSL Labs rating calculation algorithm [62]. This dynamic is characteristic of the fast-paced nature of the security ecosystem of TLS deployments. Although servers are maintained by some providers, the discovery of new vulnerabilities and the reinvention of old ones leading to new threats make constant vigilance a necessity for server administrators and (mHealth) app providers. The apparent security deterioration of servers by their grades might indicate a slower pace of app providers in keeping their TLS deployments secure.

### Common Security Concerns and Recommendations

Common security concerns are listed and summarized below. Prevention, mitigation, or alleviation recommendations are given:

1. Apps were found using a server without valid certificates both for their functional backend as well as for other purposes. This is problematic as this implies missing or erroneous certificate validation in the apps. When a client does not expect valid credentials from the server, any attacker can present equally invalid certificates and impersonate the server. This enables MitM attacks. It is strongly recommended to use a trusted CA and have a valid certificate issued for the domains that are to be secured.
2. A number of apps communicate with servers through unsecured channels. To find and prevent apps and users from using unsecured connections, a server could be configured to use HSTS. In addition, client apps should be inspected and any unsecured URL schemes should be removed.
3. Insecure server configurations indicated by a low Qualys SSL Labs’ rating were found in multiple apps’ server backends. This includes a missing set cipher order and the support of vulnerable cipher suites. These vulnerabilities can be exploited to undermine the security supplied by the use of TLS. Insecure server configurations can change over time. Any time a new exploit is discovered and/or is widely exploited, a server operator should update his server’s configuration. To keep a server configured as securely as possible, the basic security concerns should be understood [5], server-side security patches installed, and the domains should be tested using a service similar to Qualys SSL Labs’ server test [60].
4. Most apps use multiple advertising or analytics servers. Not only does this add to the data and processor time used by apps, for medical apps, but can also be especially problematic as the analytics data can undermine a user’s or patient’s privacy. A patient looking for pregnancy-related content, for example, might be pregnant. In addition, most of these services appear to be located outside the EU, and most apps used at least one such server that received a rating below the A range by Qualys SSL Labs. Although the use of advertising and analytics services is common in mobile apps, mHealth app developers should thoroughly reconsider the usage of such third-party services and frameworks [73-75]. A possible trade-off could be to offer an opt-out function to the user [3].

### Conclusions

Modern mHealth apps from popular subcategories were tested in depth and their behavior was analyzed. Although servers of mHealth apps performed significantly better than servers in general, most apps communicate with a considerable number of different servers by different operators. It was observed that these servers and connections to them are regularly not as secure as connections to the apps’ functional backends. The services behind some of these servers (advertisement and app analytics) should also be seen critically in regard to user or patient data protection. Almost half of all apps communicate with functional backends that do not offer a secure TLS setup (non-A Qualys SSL Labs rating).

The most severe problems observed in a small number of apps can expose patient communication with health care professionals, be exploited to display false or harmful information to the user, or used to send data to an app facilitating further damage to the device. These problems include communication through entirely unsecured connections, a mix of secured and unsecured connections, invalid certificates used by servers, certificate chain validation issues, missing support for modern TLS versions, and unpatched vulnerabilities.

As made evident by the comparison of the results discussed in this study with results of previous studies, the security of servers is heavily dependent on the existence and propagation of vulnerabilities. A provider of mHealth apps dealing with (potentially) sensitive information should have an even higher interest in keeping their servers up-to-date and vulnerabilities patched.

The recommendations proposed in the previous section can be used by app developers to improve their transport security setup and prevent putting patients and/or users at risk.

## Appendix

Multimedia Appendix 1 Definitions of the subcategories of apps found under the medical category.

Multimedia Appendix 2 Table containing detailed results and information about the Android apps tested.

Multimedia Appendix 3 Table containing detailed results and information about the iOS apps tested.

Multimedia Appendix 4 Table containing the numbers of connections to servers per app and security characteristic for the Android apps tested.

Multimedia Appendix 5 Table containing the numbers of connections to servers per app and security characteristic for the iOS apps tested.

Multimedia Appendix 6 App descriptions, apps by categories and summarized results from server security results of popular mHealth apps form early 2018 for both iOS and Android.

Multimedia Appendix 7 Detailed results from server security results of popular mobile health apps from early 2018 for Android.

Multimedia Appendix 8 Detailed results from server security results of popular mobile health apps from early 2018 for iOS.

## Abbreviations

API: application programming interface
ARP: address resolution protocol
BEAST: Browser Exploit Against Secure Socket Layer/Transport Layer Security
CA: certificate authority
CRIME: Compression Ratio Info-leak Made Easy
CSV: comma-separated value
DNS: domain name system
DOS: denial-of-service
EC: elliptic curve
ECDHE: Elliptic Curve Diffie Hellman Ephemeral
EU: European Union
GDPR: General Data Protection Regulation
HSTS: Hypertext Transfer Protocol Strict Transport Security
HTTPS: HTTP Secure
IETF: Internet Engineering Task Force
IP: internet protocol
KRACK: Key Reinstallation Attack
mHealth: mobile health
MitM: man-in-the-middle
OS: operating system
OWASP: Open Web Application Security Project
PKI: Public Key Infrastructure
POODLE: Padding Oracle On Downgraded Legacy Encryption
ROBOT: Return Of Bleichenbacher’s Oracle Threat
RSA: Rivest-Shamir-Adleman
SSL: Secure Socket Layer
TCP: transmission control protocol
TLS: Transport Layer Security
WEP: wired equivalent privacy
WPA: Wi-Fi Protected Access
