# Supplementary material for: Server-Focused Security Assessment of Mobile Health Apps for Popular Mobile Platforms
Source: J Med Internet Res. 2019 Jan 23;21(1):e9818. doi: 10.2196/jmir.9818 (PMC6364205; doi:10.2196/jmir.9818)
Supplement: Multimedia Appendix 1 [file jmir_v21i1e9818_app1.pdf]

Category	Definition/Criteria
Consultation, Communication & Interaction	• No need to relate to a specific domain<br>• Focus on explicit diagnostic/interpreting features (e.g. condition prediction) via interrogation/scanning<br>• Focus on features for direct consultation of a physician (e.g. (video) chat), establishing/facilitating communication a physician (e.g. contact register) and likewise
Drug Information, Shopping & Compliance	• Specifically related to the drug domain<br>• Specifically addressing individuals, not health professionals per se<br>• Focus on features for drug information retrieval, drug shopping, therapy compliance tracking features (e.g. patient information leaflet register, drugstore, intake tracking) and likewise
Fertility, Pregnancy & Parenthood	• Specifically related to the procreation domain<br>• Specifically addressing individuals, not health professionals per se<br>• Focus on features for tracking of fertility-, pregnancy- or parenthood-related data (e.g. birth control, cycle tracking, child development) and likewise
Health, Fitness & Monitoring	• No need to relate to a specific domain<br>• Specifically addressing individuals, not health professionals per se<br>• Focus on features for tracking of vital signals or mental feeling (e.g. blood pressure, mood), provision/monitoring of fitness- or health-related exercise (e.g. stress relieve) and likewise
Reference & Learning	• No need to relate to a specific domain<br>• Addressing individuals as well as health professionals<br>• Focus on features for provision of information on a specific topic for health professionals (e.g. drug indication during pregnancy), official information for individuals (e.g. vaccination), medicine-related e-learning (e.g. nursing education flashcard set) and likewise
Medical Technology (not selected)	• Specifically related to the medical technology domain<br>• Focus on features for control/monitoring of medical technology (e.g. hearing aid) and likewise
Other (not selected)	• Medical context but not categorizable as one of the above
Non-Medical (not selected)	• No medical context (e.g. teeth whitening in photography)
