# Supplementary material for: Server-Focused Security Assessment of Mobile Health Apps for Popular Mobile Platforms
Source: J Med Internet Res. 2019 Jan 23;21(1):e9818. doi: 10.2196/jmir.9818 (PMC6364205; doi:10.2196/jmir.9818)
Supplement: Multimedia Appendix 6 [file jmir_v21i1e9818_app6.pdf]

Multimedia Appendix

The following tables list, describe, show the category distribution of, and summarized the results for the apps selected for tests performed in early 2018.

Top-lists for the 'medical' category of free Android and iOS apps from appannie.com retrieved on December 20, 2017:

iOS

#	Name	Publisher	Function	Assigned Category	both platforms
1.	Schwangerschaft+	Health & Parenting Ltd.	Personalized pregnancy information & tracking	pregnancy/fertility related	yes
2.	myPill Pillen Erinnerung	Bough	Birth control reminder	pregnancy/fertility related	
3.	DocCheck Flexikon	DocCheck Medical Services	Public collection of medical data, like Wikipedia, editable by users	reference/learning	yes
4.	Baby +	Health & Parenting Ltd.	Personalized pregnancy information & tracking	pregnancy/fertility related	yes
5.	Ada	Ada Health	Health, symptom tracker, can suggest diagnostis based on input	consulting/communication	yes
6.	Moodpath App - Depression, Burnout & Anxiety Test	MoodPath UG	Interactive two-week depression and anxiety screening program	consulting/communication	yes
7.	PillReminder – Denk an mich	Sanofi	Birth control reminder	pregnancy/fertility related	
8.	Arznei aktuell	ifap GmbH	Comprehensive drug database, drug interaction information, treatment information etc.	drug information	yes
9.	Arztsuche jameda	jameda GmbH	Finding doctors near the user	consulting/communication	

			and listing ratings, contact details etc.		
10.	iMamaiPapa	GesundBleiben	Personalized pregnancy information & tracking	pregnancy/fertility related	
11.	Pillenalarm	Jenapharm GmbH & Co. KG	Birth control reminder	pregnancy/fertility related	
12.	Babyentwicklung im 1. Jahr	Kigorosa	Personalized pregnancy information & tracking	pregnancy/fertility related	
13.	Embryotox	Beratungszentrum Embryonaltoxikologie	Drug information specifically during pregnancy and the time after; targeted at professionals	pregnancy/fertility related	
14.	I care WISSEN TO GO	Georg Thieme Verlag KG	Knowledge collection targeted at health care professionals during their apprenticeship	reference/learning	yes
15.	DocCheck Help	DocCheck Medical Services	Finding and communicating with doctors (payed)	consulting/communication	yes
16.	BC Pillen Erinnerung	Sebastian Lang	Birth control reminder	pregnancy/fertility related	
17.	Schwangerschaft Lebensmittelampel	Kigorosa	Food database with special pregnancy related information	pregnancy/fertility related	
18.	BlutdruckDaten	Horst Klier	Track, monitor and store blood pressure	health/fitness	yes
19.	PROMETHEUS – LernKarten	Georg Thieme Verlag KG	Personalized pregnancy information & tracking	reference/learning	
20.	Blutspende	deltacity.net	Information about blood donations	consulting/communication	

Android

#	Name	Publisher	Function	Assigned Category	both platforms
1.	Kindernotfall-App	BARMER	Provides advice in emergency situations for parents	consulting/communication	
2.	Univadis	Aptus Health International, Inc.	News and seminar information for health care professionals	others	
3.	Schwangerschaft+	Health & Parenting Ltd.	Personalized pregnancy information & tracking	pregnancy/fertility related	yes
4.	Ada	Ada Health	Health, symptom tracker, can suggest diagnosis based on input	consulting/communication	yes
5.	Periodenkalendar	Amila	Birth control reminder	pregnancy/fertility related	
6.	Moodpath App - Depression, Burnout & Anxiety Test	MoodPath UG	Interactive two-week depression and anxiety screening program	consulting/communication	yes
7.	Menstruations-Kalendar	Emily Powell	Birth control reminder	pregnancy/fertility related	
8.	mySugr: the blood sugar tracker made just for you	mySugr GmbH	Health tracker specialized on diabetes patients, tracking blood glucose levels	health/fitness	
9.	Lady Pill Reminder	Sergio Viudes	Birth control reminder	pregnancy/fertility related	
10.	Baby +	Health & Parenting Ltd.	Personalized pregnancy information & tracking	pregnancy/fertility related	yes
11.	BlutdruckDaten	Horst Klier	Track, monitor and store blood pressure	health/fitness	yes
12.	Disorder & Diseases Dictionary	ufostudio	Disorder and disease database, available offline	reference/learning	
13.	Diagnosia Arzneimittel App Deutschland	Diagnosia	Comprehensive drug database, drug interaction information, treatment information etc. for health care professionals	drug information	
14.	DocCheck Flexikon	DocCheck Medical Services	Public collection of medical data,	reference/learning	yes

			like Wikipedia, editable by users		
15.	DocCheck Help	DocCheck Medical Services	Finding and communicating with doctors (payed)	consulting/communication	yes
16.	Medikamente-per-klick	Luitpold-Apotheke	Online drug store	others	
17.	Arznei aktuell	ifap GmbH	Comprehensive drug database, drug interaction information, treatment information etc.	drug information	yes
18.	Apotheke vor Ort	Wort und Bild Verlag	Drug reservation (e.g. by sending a photo of a prescription), interaction with local drug stores, drug store information, drug information, treatment information, drug interaction checks, special interest news (drugs), information about alternative medical treatment, encyclopedia for medical terms	drug information	
19.	I care WISSEN TO GO	Georg Thieme Verlag KG	Knowledge collection targeted at health care professionals during their apprenticeship	reference/learning	yes
20.	Ovy - Period, ovulation, nfp, bbt, menstruation	Ovy	Birth control reminder	pregnancy/fertility related	

Category distribution of the Apps:

Assigned category	Android, n	iOS, n	Sum	Sum of distinct apps across iOS and Android
Pregnancy or Fertility related	6	10	16	14
Drug information	3	1	4	3
Reference or Learning	3	3	6	4
Consulting or Communication	4	5	9	6
Health and Fitness	2	1	3	2
Others	2	0	2	2
Total	20	20	40	31

Summarized results

Results for the apps. The results list 'unique' apps. The earlier research regarded apps that were available on both iOS and Android as a single app in this category.

No.	Security issues	Android (functional) n=20	iOS (functional) n=20	Android (others) n=20	iOS (others) n=20	Total (functional) n=40	Total (others) n=40	Total (functional) unique apps n=31	Total (others) unique apps n=31
1.	SSL Labs Non-A rating	3	8	13	12	11	25	8	19
2.	Server only offers TLS version < 1.2	2	3	0	0	5	0	3	0
3.	Server without set cipher order	0	3	1	3	3	4	3	4
4.	Certificate (chain) validation issues present	0	2	4	2	2	6	2	5
5.	Downgrading Vulnerabilities	2	4	13	12	6	25	4	19
6.	Servers outside the EU	7	7	20	16	14	36	11	29
7.	Missing forward secrecy support	2	2	0	0	4	0	2	0
8.	HSTS support	1	4	14	14	5	28	10	21
